# A national study of plasma use in critical care: clinical indications, dose and effect on prothrombin time

**DOI:** 10.1186/cc10129

**Published:** 2011-04-05

**Authors:** Simon J Stanworth, Timothy S Walsh, Robin J Prescott, Robert J Lee, Douglas M Watson, Duncan Wyncoll

**Affiliations:** 1 Department of Haematology/Transfusion Medicine, John Radcliffe Hospital, NHS Blood & Transplant/Oxford Radcliffe Hospitals Trust, and University of Oxford, Osler Road, Headington, Oxford, OX3 9BQ, UK; 2 Department of Critical Care, Royal Infirmary of Edinburgh, Little France Crescent, Edinburgh, EH16 4SB, UK; 3 Centre for Population Health Sciences, University of Edinburgh, Teviot Place, Edinburgh, EH8 9AG, UK; 4 Scottish National Blood Transfusion Service, 25 Shelley Road, Gartnavel, Glasgow, G12 0XB, UK; 5 Department of Critical Care, Guy's & St Thomas' NHS Foundation Trust, Lambeth Palace Road, St Thomas's Hospital, London, SE1 7EH, UK

## Abstract

**Introduction:**

Fresh frozen plasma (FFP) is widely used, but few studies have described patterns of plasma use in critical care. We carried out a multicentre study of coagulopathy in intensive care units (ICUs) and here describe overall FFP utilisation in adult critical care, the indications for transfusions, factors indicating the doses used and the effects of FFP use on coagulation.

**Methods:**

We conducted a prospective, multicentre, observational study of all patients sequentially admitted to 29 adult UK general ICUs over 8 weeks. Daily data throughout ICU admission were collected concerning coagulation, relevant clinical outcomes (including bleeding), coagulopathy (defined as international normalised ratio (INR) >1.5, or equivalent prothrombin time (PT)), FFP and cryoprecipitate use and indications for transfusion.

**Results:**

Of 1,923 admissions, 12.7% received FFP in the ICU during 404 FFP treatment episodes (1,212 FFP units). Overall, 0.63 FFP units/ICU admission were transfused (0.11 units/ICU day). Reasons for FFP transfusion were bleeding (48%), preprocedural prophylaxis (15%) and prophylaxis without planned procedure (36%). Overall, the median FFP dose was 10.8 ml kg^-1^, but doses varied widely (first to third quartile, 7.2 to 14.4 ml kg^-1^). Thirty-one percent of FFP treatments were to patients without PT prolongation, and 41% were to patients without recorded bleeding and only mildly deranged INR (<2.5). Higher volumes of FFP were administered when the indication was bleeding (median doses: bleeding 11.1 ml kg^-1^, preprocedural prophylaxis 9.8 ml kg^-1^, prophylaxis without procedure 8.9 ml kg^-1^; *P *= 0.009 across groups) and when the pretransfusion INR was higher (ranging from median dose 8.9 ml kg^-1 ^at INR ≤1.5 to 15.7 ml kg^-1 ^at INR >3; *P *< 0.001 across ranges). Regression analyses suggested bleeding was the strongest predictor of higher FFP dose. Pretransfusion INR was more frequently normal when the transfusion indication was bleeding. Overall, posttransfusion corrections of INR were consistently small unless the pretransfusion INR was >2.5, but administration during bleeding was associated with greater INR corrections.

**Conclusions:**

There is wide variation in FFP use by ICU clinicians, and a high proportion of current FFP transfusions are of unproven clinical benefit. Better evidence from clinical trials could significantly alter patterns of use and modify current treatment costs.

## Introduction

Fresh frozen plasma (FFP) and cryoprecipitate are used widely in hospital practice across a range of clinical specialties, especially in critical care units [1-5]. FFP is prescribed for two main indications: first, to prevent bleeding (prophylaxis), and second, to stop bleeding (therapeutic). However, there are few detailed, prospective, descriptive data from large studies describing these patterns of use in the critically ill. FFP is associated with well-recognised risks [6,7], some of which are probably underrecognised, such as transfusion-associated lung injury and transfusion-associated circulatory overload [8-13]. Moreover, recent concerns that red cell transfusion may adversely affect clinical outcomes raises concerns that other blood components may not always benefit patients [14-17].

We previously reported a large national study of coagulopathy in UK intensive care units (ICUs), which showed that 30% of all critical care admissions had at least one episode of prothrombin time (PT) prolongation [18]. We described risk factors for the development of coagulopathy and showed an association with higher ICU mortality, but did not describe the use of FFP in detail. The aim of this analysis of the study database was to describe plasma use in detail, with the following research questions. (1) What reasons do clinicians give for administering FFP, and how does the presence of bleeding and coagulopathy influence the dose? (2) What PT or international normalised ratio (PT/INR) triggers are currently used by clinicians, and how do these relate to the doses administered? (3) What changes in PT/INR occur following FFP administration? We also describe the current use of cryoprecipitate in UK critical care, since it is often prescribed with or as an alternative to FFP.

## Materials and methods

A detailed description of the Intensive Care Study of Coagulopathy (ISOC study) has been previously published [18]. ISOC was a prospective observational cohort study of sequential patients admitted to 29 ICUs in Scotland, England, Northern Ireland and Wales. ICUs were mixed general units and did not include specialist cardiac centres. All admissions were eligible for inclusion, with the exception of readmissions during the same hospital stay and patients with short life expectancy (under 4 hours). The study was observational, and only routinely available clinical data were recorded. The ethics committee waived the need for patient consent (Lothian Research Ethics Committee).

Data were collected prospectively from patient records for the 24 hours prior to admission and every subsequent day in the ICU until discharge, death or 30 days (for patients remaining in the ICU at this time). Data collected included haematology and coagulation test results, all procedures, the occurrence of clinically significant haemorrhage during each 24-hour period (defined as estimated total cumulative blood loss >300 ml, 1 U of red cells or bleeding from a critical site, such as intracranial, as defined in the Audit of Transfusion in Intensive Care in Scotland study [19]), and all blood component transfusions. The timing of FFP transfusion was recorded, and the reasons for FFP were classified by clinical staff into four categories: 'coagulopathy with bleeding', 'coagulopathy with no bleeding', 'coagulopathy with no bleeding prior to an invasive procedure' or 'other'. An additional data collection form was completed on the first day that a patient developed laboratory evidence of coagulopathy (defined below) and included a range of variables predefined by consensus by the investigators as potential factors that might influence the occurrence of coagulopathy.

All data were scanned into a database using the ReadSoft system ('Eyes and Hand', Readsoft Ltd, Milton Keynes, UK), and quality control procedures included computer-based sense checking, manual checking of all queries and resolution of data queries by research staff in individual ICUs when necessary.

The definition of coagulopathy was based on a laboratory threshold INR value >1.5 or an equivalent PT beyond local reference range. Around one-half of participating ICUs reported results as PTs and one-half reported them as INRs. We therefore converted data to an INR value using the local International Specificity Index (ISI) for the laboratory, which enabled comparison across all ICUs.

### Analysis

We have previously described in detail the epidemiology of PT prolongation for the cohort, including prevalence, risk factors, severity and relationship to ICU mortality [18]. The present analysis is focussed on a detailed description of FFP use, but we also describe the use of cryoprecipitate as, in the UK, both components tend to be used to manage PT prolongation. Prothrombin complex concentrates and fibrinogen concentrate are not in routine clinical use for the management of coagulopathy in the critically ill.

#### Use of FFP

We calculated the proportion of patients who received FFP during their ICU stay and the number of units transfused. Overall FFP use in critical care was calculated per ICU admission and per ICU day. The duration of an episode of PT prolongation was defined as the time between the first recorded point when the INR was >1.5 until the first recorded INR ≤1.5 without FFP transfusion support for 12 hours. An FFP treatment episode was defined as including all units of FFP where the time between starting the transfusion of successive units was 2 hours or less. FFP transfusion episodes were reported per patient and assessed by the INR values preceding transfusion and by the degree of correction up to 6, 12 or 24 hours after transfusion. We used the estimated patient weight recorded for each patient to calculate the dose of FFP administered for each FFP transfusion episode in milligrams per kilogram.

#### Dose of FFP and reason for administration

We plotted the distribution of dose of FFP administered as a histogram and calculated the median (first to third quartiles) dose administered overall, and according to the INR value preceding transfusion and the clinical indication given for transfusion. We used three approaches to describe the reasons for FFP transfusion episodes. First, we used the reason recorded at the time of FFP transfusion by clinicians (see above). Second, we used the binary data field recorded each day, asking whether a clinically significant haemorrhage had occurred to classify each FFP transfusion episode as associated with bleeding or not. Third, each FFP transfusion episode was categorised according to whether it occurred either in a patient who had no episodes of PT prolongation documented during ICU stay, or during an episode of PT prolongation or outside an episode of PT prolongation but in a patient who experienced PT prolongation at some other time during their ICU stay.

We calculated the volume of FFP used for each of the above-defined categories and presented these data as medians (first to third quartiles) for volume, FFP units and volume per estimated patient weight. The Kruskal-Wallis test was used to compare FFP use for the various approaches for classifying the reason for FFP transfusion. A regression model was also undertaken, using FFP volume as the dependent variable, to explore the relative association of the three methods described above for categorising reasons for FFP transfusion simultaneously, and allowing for possible correlation within patients.

#### INR values pre-FFP transfusion

We used the INR preceding each FFP transfusion episode as an indicator of the "INR trigger" and recorded the frequency in ranges of INR for all FFP transfusion episodes for each of the clinical indication categories and for FFP transfusions administered on days with significant bleeding or no bleeding. INR values were compared across these categories using χ^2 ^tests. The volume of FFP transfusion in relation to the INR preceding transfusion was calculated and compared using χ^2 ^tests.

#### Change in INR post-FFP transfusion

We calculated the proportion of patients who had an INR value rechecked within 24 hours of FFP transfusion, and the distribution of times from FFP transfusion to rechecking coagulation status. For FFP transfusions with a repeat INR recorded within 24 hours, we calculated the change from pre- to post-FFP transfusion to describe the correction of INR that occurred using the pretransfusion INR to assess change, assuming a value was recorded during the 12 hours prior to FFP transfusion. We explored changes in INR values for different pretransfusion INR values. To do this with appropriate adjustment for potential confounding, an analysis was performed to model the change in INR level in relation to the variables of pretreatment INR, dose of FFP, indication for treatment and time after transfusion to INR measurement using the following approach. In an initial stage, the effect of regression to the mean of INR levels was allowed for. We acknowledged that pretreatment INR might be associated with the indication for treatment and FFP dose, so, to avoid confounding, the pretreatment INR was initially regressed on these variables and the residuals were used to produce an adjusted pretreatment INR. The posttreatment INR was then regressed on the adjusted pretreatment level to allow for regression to the mean. The residuals from this regression, when added to the mean posttreatment INR, gave the adjusted posttreatment INR, or, when added to the mean change in INR, gave the adjusted change in INR. This in turn was regressed on the dose of FFP, the indication for treatment and the time after infusion to performing INR measurement to produce the required analysis.

#### Cryoprecipitate use

We calculated the proportion of patients who received cryoprecipitate during the 24 hours prior to and during ICU stay. The distribution of cryoprecipitate use was evaluated by indication and for patients with documented bleeding. Transfusions of cryoprecipitate were assessed by fibrinogen values preceding transfusion and by the degree of correction up to 24 hours after transfusion.

## Results

### Use of FFP

Of 1,923 admissions during the study period, 12.7% (244 patients) received FFP in the ICU. These patients received 404 FFP treatment episodes comprising 1,212 units of FFP (or an average 5 U of FFP/transfused patient). For all ICU admissions, this represented a rate of 0.11 U of FFP/ICU day and 0.63 U of FFP/ICU admission. A total of 576 (30%) patients had episodes of coagulopathy defined by PT prolongation, of whom 188 (33%) received transfused FFP. These patients received 86% (1,037 of 1,212) of the total FFP units administered during the study period.

### Dose of FFP and reason for administration

**Figure 1:**
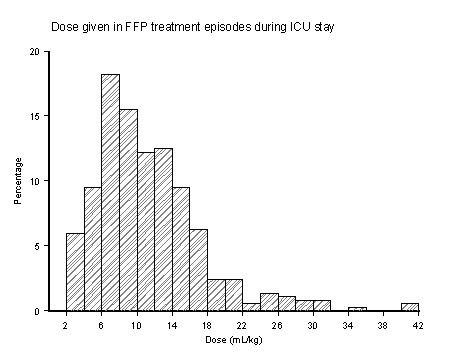
**Doses of fresh frozen plasma administered**. Graphed histogram results summarising the range of doses of fresh frozen plasma (FFP) given in treatment episodes during intensive care unit (ICU) stay.

A wide range of FFP treatment doses were administered (Figure 1). Overall a median (first to third quartile; range) of 803 ml (541 to 1,093; 220 to 2,775 ml) were administered per episode, which equated to a median 10.8 ml kg^-1 ^(7.2 to 14.4; 2.4 to 41.1 ml kg^-1^). A proportion of 69% (277 of 404) of FFP treatments were administered during episodes of PT prolongation, comprising 71% (856 of 1,212) of the total FFP transfused. A significant proportion (31%) was administered to patients who had no PT prolongation at the time of transfusion.

A clinical indication for FFP transfusion was recorded for 388 (96%) of 404 FFP treatment episodes. The reason was documented as bleeding for 48% of episodes, prophylaxis for a procedure for 15%, and in 36% of cases no procedure was planned and no bleeding was documented as being present. Six treatments were given for other reasons (1.5%). The doses of FFP administered in relation to the three approaches to classifying the circumstances of administration are shown in Table 1. On the basis of the reasons given by clinicians for administering FFP, on average 1 FFP unit more (approximately 300 ml) was administered when clinicians stated the indication was bleeding compared with a nonbleeding indication (*P *< 0.0001). On the basis of the association with recorded clinically significant bleeding on daily charts, haemorrhage was recorded in 258 (13%) of 1,923 patient admissions on a total of 410 patient days, and most frequently affected the gastrointestinal tract, chest, and abdominal cavity. Clinically significant haemorrhage was recorded on the day of FFP transfusion for 47% of FFP transfusion episodes, which was virtually identical to the clinical classification documented. This analysis confirmed that when bleeding was present, on average, 1 FFP unit more (300 ml; 2 ml kg^-1^) was administered than when no bleeding was documented (Table 1) (*P *< 0.0001). This difference was even more marked when all FFP treatments administered on a calendar day on which bleeding was documented were included (median FFP dose 14.4 ml kg^-1 ^(9.1 to 24.9; range, 2.4 to 54.8). When FFP use was classified according to the presence of prolongation of INR, higher volumes of FFP were administered during episodes of INR prolongation compared with transfusions given at other times or to patients who never had documented INR prolongation (Table 1).

**Table 1:** FFP administered in relation to the three approaches to classifying the circumstances of administration^a^

Variable	FFP units (median, first to third quartile)	FFP volume, ml (median, first to third quartile)	FFP dose, ml kg^-1^ (median, first to third quartile)
Reason given by clinician at time of FFP transfusion			
Coagulopathy with bleeding (*n *= 185)	3 (2 to 4)	875 (558 to 1104)	11.1 (7.8 to 15.3)
Coagulopathy without bleeding (*n *= 138)	2 (2 to 4)	561 (509 to 854)	8.9 (6.8 to 13.1)
No bleeding prior to procedure (*n *= 59)	2 (2 to 4)	560 (528 to 1017)	9.8 (6.9 to 13.0)
*P *value for differences across groups	<0.0001	<0.0001	0.009
All (*N *= 404)	3 (2 to 4)	736 (536 to 1092)	10.2 (7.0 to 14.1)
			
Clinically significant haemorrhage recorded on day of transfusion			
No (*n *= 215)	2 (2 to 4)	561 (517 to 1017)	9.1 (6.7 to 13.2)
Yes (*n *= 189)	3 (2 to 4)	875 (558 to 1100)	11.1 (7.9 to 14.7)
*P *value for differences across groups	<0.0001	<0.0001	0.006
			
Use of FFP in relation to occurrence of PT prolongation during ICU admission			
FFP administered but no PT prolongation occurred at any time during ICU admission (*n *= 64)	2 (2 to 4)	568 (502 to 936)	8.5 (5.7 to 12.6)
PT prolongation occurred during ICU admission, but FFP administered out with an episode of PT prolongation (*n *= 63)	2 (2 to 4)	570 (535 to 1077)	8.6 (6.3 to 13.7)
FFP administered during an episode of PT prolongation (*n *= 277)	3 (2 to 4)	806 (542 to 1092)	10.8 (7.3 to 14.4)
*P *value for differences across groups	0.11	0.037	0.014

^a^FFP, fresh frozen plasma; PT, prothrombin time; ICU, intensive care unit.

In the regression model, using volume given as the dependent variable and allowing for all of these three categorisations simultaneously and for possible correlation within patients, there was no interaction between these factors in predicting the FFP volume administered. In an additive model, the reason given by the clinician (bleeding) had the strongest association with the FFP volume administered (*P *= 0.02), with weaker associations found with the relationship to coagulopathy (*P *= 0.08) and occurrence of haemorrhage on the day of transfusion (*P *= 0.20).

### INR values pre-FFP transfusion

An INR value preceding FFP transfusion (within 12 hours) was available for 90% (362 of 404) of episodes and varied widely as reported for INR (Table 2). A high proportion of all FFP treatments were administered to patients without clinical evidence of bleeding and with either no or minor INR prolongation prior to transfusion (Table 2). For example, 40% of all FFP treatments were given to patients without recorded bleeding whose preceding INR was <2.5, and for 8.6% of all treatments there was no haemorrhage and the preceding INR was <1.5. When we used recorded, clinically significant haemorrhage on the day of FFP transfusion to dichotomise the population, a similar pattern was seen: 41% of FFP transfusions were given to patients with no bleeding on the day of transfusion and a pretransfusion INR of <2.5, and 8% were given to patients with INR <1.5.

**Table 2:** INR preceding (within 12 hours) FFP treatment during ICU stay for all admissions^a^

	INR				
	
Variable	≤1.5, *n *(%)	1.6 to 2.5, *n *(%)	2.6 to 3.5, *n *(%)	3.6 to 5.0, *n *(%)	>5.0, *n *(%)
All episodes, *n *(%)	126 (34)	187 (51)	30 (8)	14 (4)	9 (2)
Reason for FFP transfusion recorded by clinician					
Coagulopathy with bleeding	73 (42)	86 (50)	8 (5)	2 (1)	4 (2)
Coagulopathy without bleeding	32 (26)	65 (52)	15 (12)	9 (7)	4 (3)
Coagulopathy without bleeding, prior to invasive procedure	13 (25)	29 (56)	7 (13)	2 (4)	1 (2)
Clinically significant haemorrhage recorded on same day as FFP transfusion					
No	54 (29)	95 (51)	20 (11)	12 (6)	6 (3)
Yes	72 (40)	92 (51)	10 (6)	2 (1)	3 (2)

^a^INR, international normalised ratio; FFP, fresh frozen plasma; ICU, intensive care unit.

Comparing FFP transfusions for which the clinical indication was recorded as coagulopathy plus bleeding with the nonbleeding categories indicated that pretransfusion INR values were more frequently either normal or only mildly prolonged when bleeding was present (*P *= 0.006; χ^2 ^test). A similar pattern was observed when FFP transfusions administered on days with clinically significant haemorrhage were compared with those administered on days with no recorded haemorrhage (*P *= 0.002). These data show that although a high proportion of FFP treatments associated with bleeding were preceded by normal or mildly prolonged INR, as might be expected from clinical practice, this was also a feature of prophylactic or preprocedural transfusions.

Smaller doses of FFP were administered to patients when the transfusion was given outside episodes of PT prolongation (median 570 ml) or in patients who never had PT prolongation (median 568 ml) (Table 1). The dose of FFP administered in relation to increasing pretransfusion INR is shown in Table 3, which confirmed the trend for FFP dose to increase with higher INR value. There were 56 patients who received 64 FFP treatments in the ICU but never had documented INR prolongation at any stage. Clinical factors for this group have been reported previously [18]. To also explore the potential importance of activated partial thromboplastin time (APTT) values, we extracted the APTT value prior to FFP transfusion. For 24 of these 64 treatments, no APTT value was measured during the 24 hours prior to FFP administration, and for the other 40 treatments, APTT was measured but was only mildly prolonged in most cases (median APTT value 42 seconds (interquartile range 33.5 to 51.5 seconds)).

**Table 3:** Median (first to third quartile) FFP dose (ml/kg) administered for different pretransfusion INR values^a^

INR value preceding FFP treatment	Median FFP dose per treatment, ml/kg (first to third quartile)
≤1.5	8.9 (5.8 to 13.9)
1.6 to 2.0	9.7 (6.7 to 15.3)
2.1 to 2.5	13.8 (8.6 to 18.7)
2.6 to 3.0	13.7 (7.8 to 24.2)
>3.0	15.7 (11.4 to 22.1)

^a^FFP, fresh frozen plasma; INR, international normalised ratio. Results for FFP dose administered by level of pretransfusion INR are shown. *P *< 0.001 across the groups (Kruskal-Wallis test).

### Change in INR following FFP transfusion

**Figure 2:**
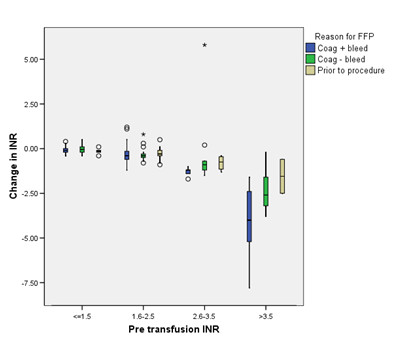
**Effects of fresh frozen plasma on changes in international normalised ratio**. Boxplot summarising the changes in international normalised ratio (INR), which are shown as medians (horizontal line), first and third quartiles (squares) and ranges (vertical lines) with outliers indicated by small circles or asterisks. The results are reported by reason for fresh frozen plasma (FFP) transfusion recorded by clinician (coagulopathy with bleeding, coagulopathy without bleeding or coagulopathy without bleeding prior to invasive procedure) for different levels of pretransfusion INR.

A posttransfusion INR was available within 6 hours for 68% (*n *= 276) of FFP transfusions (76% when the reason for transfusion was bleeding, 62% when bleeding was not recorded as the reason and 56% when the reason was prior to invasive procedures). Ninety-three percent (*n *= 375) of patients receiving FFP had a repeat INR checked within 24 hours. For FFP transfusions with a repeat INR available within 6 hours, the pre- to posttransfusion INR changes are illustrated in Figure 2. As expected, the median reductions in INR were greater when the pre-FFP transfusion values were higher (median change -0.1, -0.4, -1.0 and -2.5 for pretransfusion INRs in the ranges 1 to ≤1.5, 1.6 to 2.5, 2.6 to 3.5 and >3.5, respectively).

In univariate analyses, we found no relationship between degree of change of INR and dose of FFP used (*P *= 0.86). In the multivariable regression analysis designed to allow for regression to the mean, which included changes in INR across the three clinical categories ('coagulopathy with bleeding', 'coagulopathy with no bleeding' and 'coagulopathy with no bleeding prior to an invasive procedure') in relation to the dose of FFP administered, and taking into account relationships with pretransfusion INR, we found that the posttransfusion INRs were 0.2 (SE ±0.05) lower when FFP was given for bleeding compared to the other clinical indications.

The INR was found to rise significantly with time following the first posttransfusion INR, with a mean increase of 0.3 (SE ±0.1) over a 24-hour period.

### Use of cryoprecipitate

Transfusion of cryoprecipitate during ICU stay was recorded in only 62 of 1,923 admissions (3%) and was more frequent in admissions with INR prolongation (50 (9%) of 576 vs. 12 (1%) of 1,347). These 62 patients received 69 cryoprecipitate transfusions, most of which (46 patients; 67%) were associated with clinically significant bleeds on the same day or in the previous 24 hours. FFP was administered on 41 of these 46 occasions and was also administered on 17 of the 23 transfusion days that were not associated with bleeding. Pre- and postcryoprecipitate transfusion fibrinogen values were available for 35 of the 46 patients in whom haemorrhage was documented. The mean fibrinogen concentration prior to cryoprecipitate transfusion was 1.62 g/l (SD ±1.06) and increased to a mean of 2.89 g/l (SD ±1.67) (mean increase of 1.27 g/l (SD ±1.80)). In total, during the ICU stay, there were 7,208 fibrinogen tests on 5,970 patient-days from 1,391 patients [18]. Ninety-eight of the 7,208 fibrinogen tests performed in total gave a fibrinogen level <1 g/dl (1.4%).

## Discussion

This pragmatic national epidemiological study of admissions to general ICUs in the UK indicated that 13% of all patients admitted received FFP. There was wide variation in the dose used, even after we classified transfusion episodes according to the reasons given by clinicians, the presence of bleeding and the presence of coagulopathy defined by INR prolongation. A remarkably high proportion of FFP was administered to patients without evidence of bleeding, and many of these patients had normal or only mildly deranged INR values. For example, 40% of all FFP treatments were administered to patients without haemorrhage and in whom preceding coagulation tests were normal or only modestly deranged (INR ≤2.5), and 29% of FFP treatments were administered outside episodes of INR prolongation. Relevant clinical factors to describe the epidemiology of these subgroups of coagulopathy in critical care have been described previously [18], but our data in this paper extend these findings and illustrate the very uncertain rationale underpinning FFP administration, which must raise questions about the appropriateness of use and effectiveness of FFP.

We found that clinicians prescribed marginally higher volumes of FFP in association with bleeding and when the INR was prolonged, and our regression model found haemorrhage to be the clinical indication that was the strongest factor in predicting higher transfusion volume. However, typically only around one additional unit of FFP was administered per transfusion episode under these circumstances, with a median volume transfused of about 11 ml/kg. Current guidelines support the need for adequate doses of FFP to achieve haemostasis, typically 10 to 1 ml/kg [20,21], but even these doses of FFP may fail to correct low coagulation factor levels without taking into consideration factors such as the size of the patient, the extent of coagulation deficiency or the degree of abnormality to be corrected [2,22].

Our data suggest that under circumstances where FFP has a higher probability of patient benefit, namely, bleeding in a patient with INR prolongation, inadequate doses are currently administered to reliably correct coagulation factor deficiencies. This may explain, in part, the small decreases in INR prolongation that we observed after FFP transfusion, especially when the INR was only modestly prolonged [2,23,24]. Variable reductions in INR may also be explained by differences in the time taken for rechecking INR after FFP transfusion, although our statistical model incorporated this variable. The reasons for transfusion of inadequate volumes of FFP are unclear and require further investigation. In addition, an understanding of the nonlinear relationship between coagulation factor content and INR would also indicate why, with minimally deranged prolongations in INR, the effects of FFP transfusion aimed at raising the levels of coagulation factors and reducing the INR are likely to be very small [25]. If inadequate FFP administration influences clinical outcomes during bleeding, as is suggested by emerging evidence relating to trauma-associated coagulopathy and massive haemorrhage [26,27], the variation and inconsistent practice we have documented could affect clinical outcomes.

Conversely, the large number of FFP transfusions administered to nonbleeding patients without INR prolongation or with minor derangements suggests the possibility of widespread and inconsistent use of FFP without strong clinical rationale. Recent systematic reviews indicate the poor quality of existing evidence, including the poor predictive value of standard coagulation tests, and no clear benefit of prophylactic use or to reduce bleeding related to planned procedures [28,29]. Indeed, available data suggest that many patients with isolated minor abnormalities in PT or INR have adequate individual coagulation factor levels, which would be consistent with our data [30]. Furthermore, the FFP volumes administered in these nonbleeding situations were 9 ml/kg or less in more than 50% of cases, and these doses are highly unlikely to result in consistent increases (if any) in coagulation factor levels if deficiencies do exist [2,22-25]. Our data show that critical care clinicians in the UK continue to use FFP widely in this prophylactic situation, with evidence of insignificant changes in the INR value [2,31].

Stronger evidence is needed to guide clinicians. Specifically, evidence suggesting that correction of INR prolongation is not required in the prophylactic clinical situation could significantly alter current practice and potentially reduce demand for FFP. Continuing reports of an association between FFP administration and adverse patient outcomes in patients, including the critically ill, further emphasise the requirement to improve the evidence base, especially for transfusion of FFP to nonbleeding patients [6,9-13,32-35].

Our population estimates found that 0.63 FFP units/ICU admission were utilised in UK general critical care units, which equates to approximately 63,000 units/year, based on the 100,000 patients admitted to general ICUs in the UK each year. This comprises around 21% of the total FFP used in the UK and, of note, excludes cardiac ICUs, which were not included in our study. On the basis of our estimates of the proportion used in nonbleeding patients with normal or mildly deranged INRs (<2.5), who received 40% of FFP treatments, and our observed median use for this indication of 2 FFP units/transfusion episode, we estimate that about 40,000 units are transfused each year to patients in whom the evidence base for benefit is weak. These estimates illustrate the potential impact of an improved evidence base, particularly if this evidence supports more restrictive use. The potential cost implications of introducing newer products, such as factor complex concentrates or pathogen-inactivated plasma, are also unclear in the absence of evidence of benefit for the standard product.

There are limitations to our pragmatic study. Around one-half of the participating ICUs reported coagulation results as PT and one-half reported them as INRs. The definition of coagulopathy was based on PT prolongation and a threshold INR value >1.5. This cutoff level is commonly used by clinicians and described in the literature [19,21,36], and the use of the INR allowed the data to be compared and standardised across the ICUs, which would not be possible for the PT, given that laboratories use different thromboplastins with different laboratory-specific mean normal PT and ISI. However, the PT was developed to assess coagulation factor deficiencies, and the INR was developed to standardise assessment of the response to vitamin K antagonists [36,37]. For our primary analysis, we did not relate FFP use to APTT, which could also trigger correction of coagulation, especially in relation to bleeding. To explore the potential importance of APTT values, we examined the 56 patients who received 64 FFP treatments but never had documented PT prolongation during their ICU stay, and then extracted the APTT value prior to FFP transfusion. The results did not suggest that APTT prolongation was likely to explain the widespread use of FFP when the INR was normal or mildly prolonged, although our study was not designed to answer this question.

## Conclusions

FFP continues to be widely used as a prophylactic or precautionary treatment in critically ill nonbleeding patients with mild coagulation abnormalities. Wide variation in clinical use exists in terms of indication and the dose used, which is often inadequate to reliably increase individual factor levels. Our data support the need for more research to improve the current understanding of the risk-to-benefit profile of plasma therapy, and specifically the need (if at all) for FFP or other products, especially in relation to nonbleeding patients.

## Key messages

• FFP is widely used, but there are few data from prospective studies describing patterns of plasma use in critical care.

• In this national prospective study, of 1,923 admissions to adult critical care, 12.7% received FFP in the ICU during 404 FFP treatment episodes (1,212 FFP units).

• Thirty-one percent of FFP treatments were administered to patients without PT prolongation, and 41% were given to patients without recorded bleeding and only mildly deranged INRs (<2.5).

• Overall, posttransfusion corrections of INR were consistently small unless the pretransfusion INR was >2.5.

• There is wide variation in FFP use by ICU clinicians, and a high proportion of current FFP transfusions are of uncertain and unproven clinical benefit.

## Abbreviations

APTT: activated partial thromboplastin time; FFP: fresh frozen plasma; ICUs: intensive care units; INR: international normalised ratio; ISI: International Specificity Index; PCC: prothrombin complex concentrate; PT: prothrombin time; TACO: transfusion-associated circulatory overload; TRALI: transfusion-associated acute lung injury.

## Competing interests

The authors declare that they have no competing interests.

## Authors' contributions

The study was designed by TSW, SJS, RJL, DMW and DW. Analysis was planned by all members of the writing group. The statistical analysis was performed by RJL and RJP. All members of the writing committee contributed to writing the manuscript. All authors approved the final manuscript. TSW, SJS and RJP had full access to all of the data in the study and take responsibility for the integrity of the data and the accuracy of the data analysis.
